# Spatial variations and determinants of knowledge of ovulatory period among young women in Ethiopia: a spatial and multilevel analysis using 2016 EDHS

**DOI:** 10.1186/s12905-023-02706-4

**Published:** 2023-11-08

**Authors:** Fantu Mamo Aragaw, Meron Asmamaw Alemayehu, Nebiyu Mekonnen Derseh, Muluken Chanie Agimas, Daniel Alayu Shewaye, Habitu Birhan Eshetu, Desale Bihonegn Asmamaw, Melaku Hunie Asratie, Tadele Biresaw Belachew, Wubshet Debebe Negash

**Affiliations:** 1 Department of Epidemiology and Biostatistics, Institute of Public Health, College of Medicine and Health Sciences, University of Gondar, Gondar, Ethiopia; 2 Department of Health Education and Behavioral Sciences, Institute of Public Health, College of Medicine and Health Sciences, University of Gondar, Gondar, Ethiopia; 3 Department of Reproductive Health, Institute of Public Health, College of Medicine and Health Sciences, University of Gondar, Gondar, Ethiopia; 4 Department of Women’s and Family Health, School of Midwifery, College of Medicine and Health Sciences, University of Gondar, Gondar, Ethiopia; 5 Department of Health Systems and Policy, Institute of Public Health, College of Medicine and Health Sciences, University of Gondar, Gondar, Ethiopia

**Keywords:** Ovulatory period, Youth, Spatial analysis, Multilevel analysis, Ethiopia

## Abstract

**Background:**

Knowledge of the ovulatory period enables women in avoiding and engaging in sexual intercourse either to avoid and to have pregnancy as desired. It has been reported that young people have less knowledge of the ovulatory period. There is limited evidence about the spatial variability of knowledge of the ovulatory period among young women in Ethiopia. Hence, this study aimed to assess the spatial variation and factors sociated with knowledge of the ovulatory period among youths in Ethiopia for providing geographically targeted interventions.

**Method:**

A secondary data analysis was carried out using the 2016 Ethiopian Demographic and Health Surveys with a total weighted sample of 6143 youths. Multilevel logistic regression analysis was utilized to identify factors influencing knowledge of the ovulatory period. ArcGIS version 10.7 software and Kuldorff’s SaTScan version 9.6 was used for the spatial analysis.

**Results:**

Being older youth [AOR = 1.98; 1.46, 2.70], youths having primary education [AOR = 1.70; 1.23, 2.35], youths having secondary & higher education [AOR = 2.30; 1.41, 3.74], youths whose husbands have primary education [AOR = 1.39; 1.02, 1.91], and youths who use contraception [AOR = 1.66; 1.24, 2.22] were significant predictors of knowledge of ovulatory period. Knowledge of the ovulatory period among youth had non random spatial distribution across Ethiopia, and the primary clusters of incorrect knowledge of the ovulatory period were observed in Somalia, SNNPR, Benishangul gumuz, and Gambella regions of Ethiopia.

**Conclusion:**

There was a non-random spatial pattern in the distribution of knowledge of the ovulation period among young women in Ethiopia. Age of youth, educational status, education of husband, and contraceptive use were significant predictors of knowledge of the ovulatory period among young women in Ethiopia. Hence, interventions should prioritize at-risk youths residing in regions with limited knowledge of the ovulatory period to enhance their awareness of the fertility window.

## Introduction

Ovulation is a physiological event that occurs when the dominant follicle from the ovary separates and exits into the fallopian tube [1]. The ovulatory cycle is a natural family planning method that a woman utilizes to decide whether or not to engage in sexual relations during a fertile period [2, 3]. Knowledge of the ovulatory cycle can be a healthy family planning method for making pregnancy and fertility decisions [2, 3]. Knowing the ovulatory cycle (KOC) assists women in avoiding and engaging in sexual intercourse to avoid and obtain pregnancy and improve a woman’s reproductive health [4, 5].

Fertility awareness is the knowledge about the possibility to conceive during the menstrual cycle [6]. The ability to accurately determine ovulation is required for optimal intercourse timing [7–9]. The most important aspect of a woman’s fertility awareness is accurate ovulation timing [3]. Fertility knowledge is a useful tool that enables a woman to recognize her health [10]. Furthermore, an understanding of the ovulation cycle can be useful in the diagnosis of certain medical conditions [11].

Most youths are unaware of an ovulatory cycle and end up having an unsafe abortion [12]. Non-contraceptive users who are unaware of their ovulatory period face a higher risk of experiencing unintended pregnancies [11, 13]. Adequate knowledge of the ovulatory cycle may aid in lowering the cost of unplanned pregnancy, particularly in Sub-Saharan Africa [14–17]. A lack of knowledge about the time of ovulation can lead to unwanted pregnancies and unsafe abortions among married women [6, 18, 19].

Natural family planning methods provide a viable alternative in regions where access to modern contraception is limited, such as Ethiopia [20]. It is vital to promote the use of natural family planning methods, given the increasing health risks associated with contraception and the high rate of discontinuation [20]. Knowledge of the ovulation cycle is essential prior to the use of natural family planning methods [5]. There is less awareness about contraceptive methods including natural family planning with their limited uptake in Sub-Saharan Africa [14].

Research has shown that women in the younger age group possess a lower level of knowledge about the ovulatory cycle [4, 12, 20–26]. Evidence also indicated young women having incorrect knowledge of the ovulatory cycle are more likely to report the highest proportion of unintended pregnancies, particularly in Africa [9]. Even though several studies were conducted about knowledge of the ovulatory period in Ethiopia, none of these studies have considered community level variables and they usually focus on the entire reproductive age group. It is crucial to identify factors that affect ovulatory cycle knowledge among young women at the individual and community levels using multilevel analysis to provide evidence for policymakers. Also exploring the spatial pattern is critical for identifying locations with a high proportion of poor knowledge of ovulation and designing geographically tailored evidence-based interventions in Ethiopia. As a result, the purpose of this study was to determine the spatial variation and determinants of ovulatory cycle knowledge among young women in Ethiopia.

## Methods

### Data sources, sampling procedure, and populations

Secondary data analysis was carried out using the 2016 Ethiopian Demographic and Health Survey. Ethiopia is in the Horn of Africa, between 3° and 15° north latitude and 33° and 48° east longitude. The Demographic Health Survey (DHS) is a multi-round cross-country survey that evaluates population health, with a focus on maternal and child health, as well as population health indicators of global health importance. Young aged women (15–24 years) in Ethiopia were the source of the population whereas those found in the selected Enumeration Areas (EAs) or clusters were the study population. All young aged women between 15 and 24 years who have been in the selected Enumeration areas (EAs) and whose data are recorded in the data set were included. The survey employed a two-stage stratified cluster sampling technique to select the study participants. The Ethiopian Population and Housing Census (PHC) conducted in 2007 served as the sampling frame for 84,915 enumeration areas for the EDHS 2016. The selection of EAs in each stratum was carried out separately in two stages. In the first stage, a total of 645 EAs were chosen (202 in urban areas and 443 in rural areas) after stratifying each cluster into urban and rural areas with a probability proportional to their EA size. During the second phase, a fixed number of 28 households per cluster were chosen with an equal likelihood of systematic selection from the newly constructed household listing [27]. More information on the survey can be found in the 2016 EDHS report [27]. Data were obtained from the DHS website: www.dhsprogram.com after a formal request. The individual record data set was used for this analysis, and a total weighted sample of 6,143 young aged women was included in this study.

### Variables of the study

The outcome of this study was the knowledge of the ovulatory period, which was classified into two categories of correct knowledge and incorrect knowledge. The Knowledge of the ovulation period was assessed by the respondents by asking “When is the ovulation time?”, and the responses were “during her period”, “after the period ended”, “middle of the cycle”, “before the period begins”, “at any time”, and “don’t know”. Women who answered “middle of the cycle” was considered as having correct knowledge and coded as 1 and otherwise incorrect knowledge and coded as 0 [5, 28].

The individual-level independent variables for this study were the age of the women, women’s education, husband’s education, working status, marital status, wealth index, menstruation in the last six months, contraceptive use, and media exposure. Media exposure status is created from the frequency of reading a newspaper or magazine, watching TV, and listening to the radio. If a woman answers yes to at least one question, she has been considered to have media exposure.

**Fig. 1:**
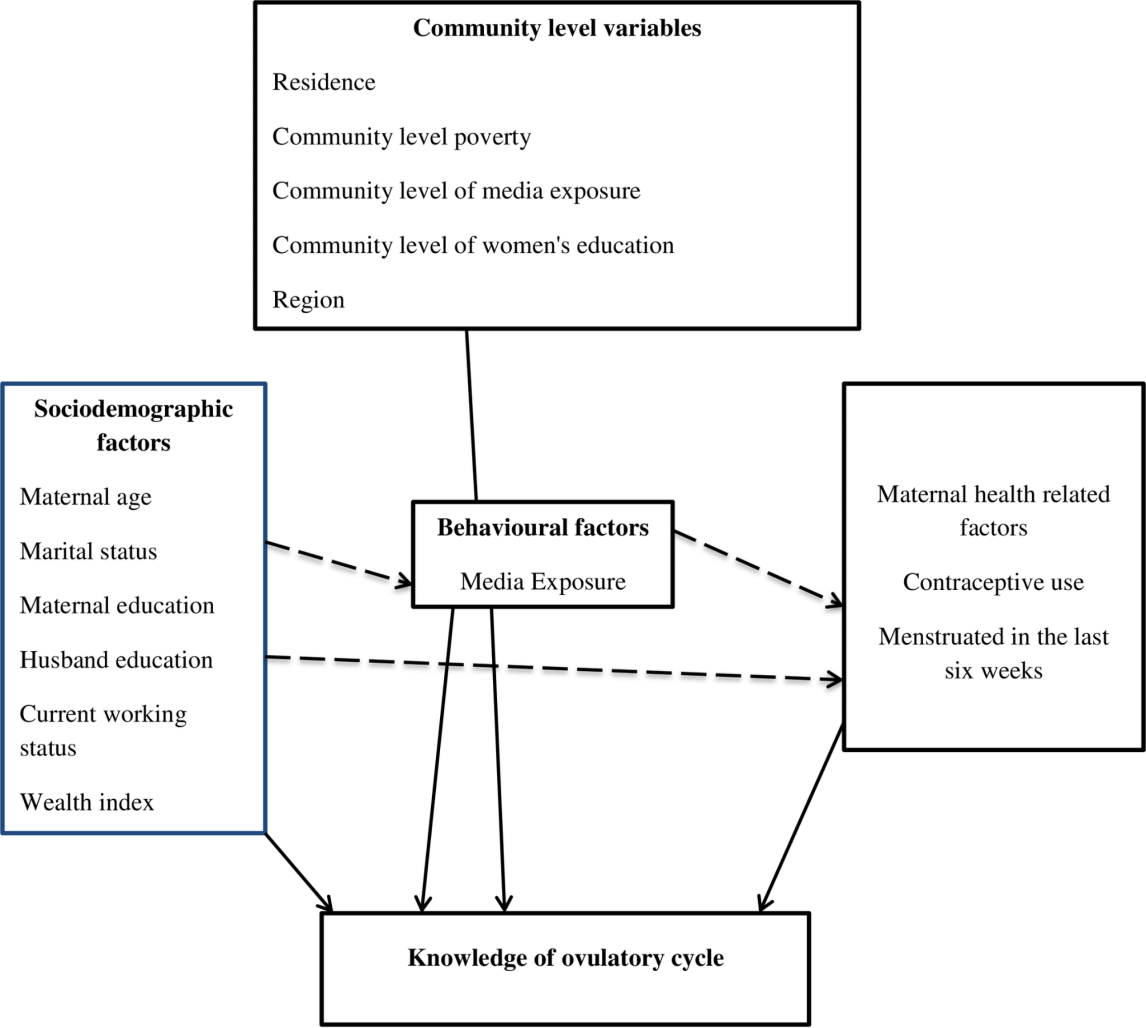
Conceptual framework of factors associated with knowledge of ovulatory cycle developed from searching of literature

As community-level factors residence, community-level poverty, community-level media exposure, community level of women’s education, and region were included. Because the data were not normally distributed, community poverty and literacy levels were classified as high or low, by using the median value as the classification cut-off point. The poverty level in a community was categorized as high if the proportion of young women in the two lowest wealth quintiles was greater than the median value, and low if the proportion was less than the median value [29]. The community level of women’s education was categorized as high if the proportion of women with at least a primary level of education was greater than the median value, and low if it was less than the median value [30]. The proportion of women who had at least one media exposure was used to assess community-level media exposure. It uses “0” to indicate low-level media coverage and “1” to indicate high-level media coverage at the community level [31]. The conceptual framework presented in Fig. 1 illustrates the relationship between knowledge of the ovulatory period and various independent variables (Fig. 1).

### Data processing and analysis

After accessing the data, it was cleaned and coded to make it suitable for analysis. Sample weights were applied to account for the strata’s unequal probability of selection which restore the survey’s representativeness. The descriptive and analytic statistic was computed using STATA version 16. We performed a multilevel logistic regression with a random effect at the cluster level, accounting for the clustered nature of the data as well as within and between community variations [32]. Variables with p-value < 0.2 in the bi-variable analysis for both individual and community-level factors were fitted in the multivariable model, and the Adjusted Odds Ratio (AOR) with a 95% CI in the multivariable model was used to declare statistical significance. The Variance Inflation Factor (VIF) was utilized to test for multicollinearity among the independent variables. The goodness of fit of our model was assessed using deviance information criteria (DIC). ArcGIS version 10.7 and SaTScan version 9.6 software were used for spatial and SaTScan analysis.

### Spatial analysis

The spatial autocorrelation (Global Moran’s Index) statistic was used to assess whether incorrect knowledge of the ovulatory period was dispersed, clustered, or randomly distributed among women aged 15–24 years in Ethiopia. The maximum peak distance at which incorrect knowledge of the ovulatory period becomes more prominent is calculated utilizing incremental spatial autocorrelation.

Getis-Ord Gi* statistics were used to identify hot and cold spots across study locations [33]. Statistical output with a high GI* denotes a “hotspot,“ whereas a low GI* suggests a “cold spot” [34]. A “hotspot” area indicates a high prevalence of incorrect knowledge of the ovulatory period while a “cold spot” area indicates a low prevalence of incorrect knowledge of the ovulatory period.

The ordinary Kriging spatial interpolation technique was used to predict areas that were not sampled based on sampled values. The spatial interpolation was performed under the assumption that spatially distributed objects are spatially correlated, and that objects that are close together are more likely to have similar properties [35, 36].

The spatial locations of statistically significant clusters for incorrect knowledge of the ovulatory period in Ethiopia were determined using Bernoulli-based model spatial scan statistics. A likelihood ratio test statistic and the p-value were utilized to decide whether the number of observed incorrect knowledge of the ovulatory period within the potential cluster was significantly higher than expected.

### Parameter estimation and model building

For the correlation of knowledge of the ovulatory period and predictor variables, the fixed effects were used to calculate the odds ratio with a 95% confidence interval and a p-value of 0.05. As a result, the following two-level multilevel model was used to describe the likelihood of having knowledge of the ovulatory period.

$$Log\left( {\frac{{\pi ij}}{{1 - \pi ij}}} \right) = {{\rm{\beta }}_{\rm{0}}}{\rm{ + \beta 1xij + \beta 2xij + \beta 3xij + \ldots \ldots }}{\rm{.uj + eij}}$$

Where πij = is the probability of knowing the ovulatory period and 1-πij represents the probability of not knowing the ovulatory period. β1xij are individual and community level variables for the ith individual in group j, respectively.

The β’s are fixed coefficients indicating a unit increase in X can result in a β unit increase in the probability of having knowledge of the ovulatory period. While the β0 represents the intercept, which is the effect on knowledge of the ovulatory period in the absence of the influence of independent variables. The uj represents the random effect, which is the community’s effect on the knowledge of the ovulatory period for the jth community, whereas eij represents random error at the individual level.

The variations between clusters were computed using Intra-Class Correlation (ICC), and the median odds ratio (MOR).The ICC demonstrates the differences between clusters in the knowledge of the ovulatory period among young women, and it is computed as ICC=$\frac{VA}{ VA+3.29}*100$, Where; VA =area-level variance [37–39]. The MOR indicates the central value of the odd ratio between the highest and the lowest risk regions when two clusters are chosen at random. The MOR is calculated as MOR=e0.95$\surd VA$, where VA = area level variance [32, 40].

In multilevel analysis, four models were fitted. The first was the null model containing no independent variables which were used to check the variability of knowledge of the ovulatory period in the community. The second (model I) hierarchical models contain individual-level variables whereas the third (model II) contains community-level variables. In the fourth model (model III) both individual and community-level variables were considered in the analysis simultaneously.

### Ethical consideration

All methods were carried out following relevant guidelines of the Demographic and Health Surveys (DHS) program. Informed consent was waived from the International Review Board of Demographic and Health Surveys (DHS) program data archivists after the consent paper was submitted to the DHS Program. The dataset was not shared or passed on to other bodies and was anonymized to maintain its confidentiality. All methods were carried out following relevant guidelines and regulations.

## Results

A total of 6,143 young aged women were included in this study. More than half of the participants 3,380 (55.04%) are in the age group of 15–19. More than three fourth of the study participants live in rural areas 4,675 (76.11%), and out of these only 20% of youths correctly know about the ovulatory period. Around 1,230 (20.03) have no education and out of these only 12.50% of youths correctly know about the ovulatory period. The prevalence of knowledge of the ovulatory period among young women in Ethiopia was 23.39% (95% CI: 22.35%, 24.47%) (Table 1).

**Table 1:** Characteristics of the study population with knowledge of the ovulatory period among young women in Ethiopia: EDHS 2016

Variables	**Categories**	Knowledge of the ovulatory period		Total weighted frequency (%)
		Yes (%) **n = 1,437 (23.40)**	No (%) **n = 4,705 (76.60)**	
Age of women	15–19	685(20.26)	2,696(79.74)	3,380 (55.04)
	20–24	752(27.24)	2,009 (72.76)	2,761 (44.96)
Marital status	Married	505 (22.71)	1,719 (77.29)	2,224 (36.21)
	Not married	932 (23.79)	2,986 (76.21)	3,918 (63.79)
Women education status	No education	153 (12.50)	1,076 (87.50)	1,230 (20.03)
	Primary	732 (21.97)	2,600 (78.03)	3,332 (54.25)
	Secondary and higher	551 (34.89)	1,029 (65.11)	1,579 (25.72)
Husband education status	No education	127 (16.93)	623 (83.07)	750 (32.65)
	Primary	242 (23.48)	788 (76.52)	1,031 (44.88
	Secondary and higher	156 (30.37)	359 (69.63)	516 (22.48)
Current working status	Not working	807 (23.39)	2,644 (76.61)	3,451 (56.19)
	Working	630 (23.41)	2,061 (76.59)	2,691 (43.81)
Menstruated in last six weeks	No	406 (20.04)	1,031 (25.05)	2,026 (32.99)
	Yes	1,620 (79.96)	3,085 (74.95)	4,116 (67.01)
Contraceptive use	Non user	1,157 (22.44)	4,002 (77.56)	5,159 (84.00)
	User	279 (28.44)	703 (71.56)	983(16.00)
Media exposure	No	531 (17.48)	2,507 (82.52)	3,039 (49.47)
	Yes	905 (29.19)	2,197 (70.81)	3,103 (50.53)
Wealth index	Poor	340 (16.77)	1,686 (83.23)	2,026 (32.98)
	Middle	227 (20.47)	886 (79.53)	1,113 (18.13)
	Rich	869 (28.96)	2,133 (71.04)	3,003(48.89)
**Community level variables**				
Residence	Urban	465 (31.68)	1,002 (68.32)	1,467 (23.89)
	Rural	972 (20.80)	3,703 (79.20)	4,675 (76.11)
Community level poverty	Low	813 (29.09)	1,983 (70.91)	2,796 (45.53)
	High	624 (18.64)	2,722 (81.36)	3,346 (54.47)
Community level of media exposure	Low	663 (18.55)	2,912 (81.45)	3,576 (58.22)
	High	773 (30.15)	1,792 (69.85)	2,566 (41.78)
Community level of women’s education	Low	581 (18.51)	2,557 (81.49)	3,138 (51.10)
	High	856 (28.50)	2,147 (71.50)	3,003 (48.90)
Region	larger central	1,240 (23.15)	4,119 (76.85)	5,360 (87.26)
	small peripherals	36 (11.21)	290 (88.79)	326 (5.32)
	metropolis	159 (35.08)	296 (64.92)	455 (7.42)

### Random effect and model comparison

In the null model, the ICC indicated that 19% of the total variability for knowledge of the ovulatory period was due to differences between clusters while the remaining unexplained 81% of the total variability in the knowledge of the ovulatory period was attributable to individual differences. The model having the lowest deviance was selected as the best-fitted model which is Model III (1991**).** The mean VIF of the final model was 1.86 which is less than five suggesting the absence of multicollinearity (Table 2**).**

**Table 2:** Parameters and model fit statistics for multilevel regression analysis models

	Null model	Model I	Model II	Model III	
**Random effect**					
VA	0.81	1.56	0.49	1.35	
ICC	0.19	0.32	0.13	0.29	
MOR	2.32	3.2	1.8	2.99	
**Model comparison**					
Deviance	6350	2201	6208	1991	
Mean VIF	__	1.43	2.13	1.86	

ICC = Inter cluster correlation coefficient, MOR =Median odds ratio, VIF: Variance inflation factor

### Spatial analysis results

#### Spatial autocorrelation of knowledge of the ovulatory period among young women in Ethiopia

**Fig. 2:**
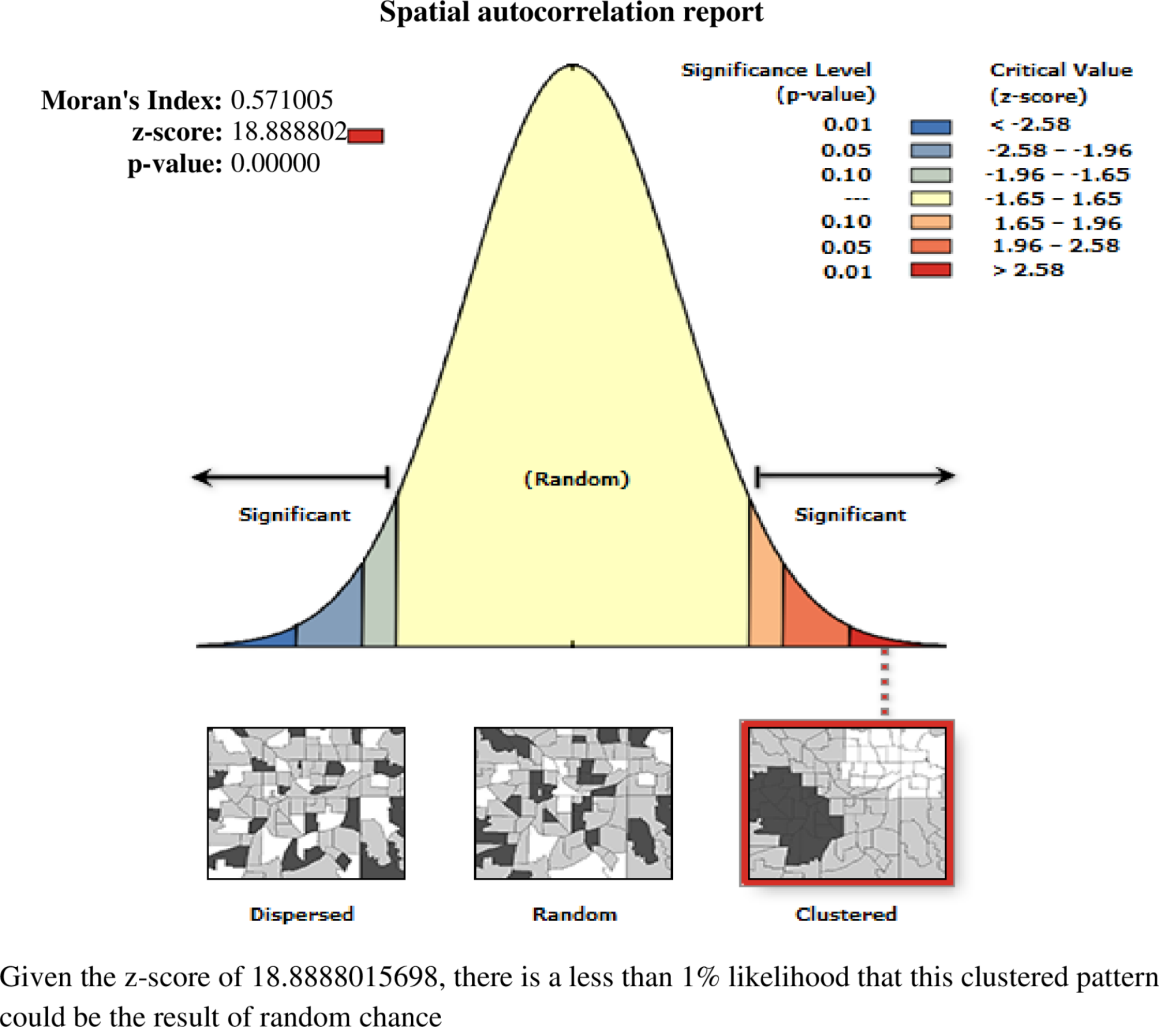
Spatial autocorrelation analysis of knowledge of the ovulatory cycle among young women in Ethiopia: EDHS 2016

The global spatial autocorrelation analysis showed a clustering pattern of knowledge of the ovulatory cycle among youths in Ethiopia (Global Moran’s I = 0.571, p value < 0.00001) (Fig. 2). The incremental autocorrelation result revealed statistically significant z-scores at a peak distance of 195.726 km 18.64 (distances; Z-score) for incorrect knowledge of the ovulatory period.

#### Hotspot analysis of knowledge of the ovulatory period among young women in Ethiopia

**Fig. 3:**
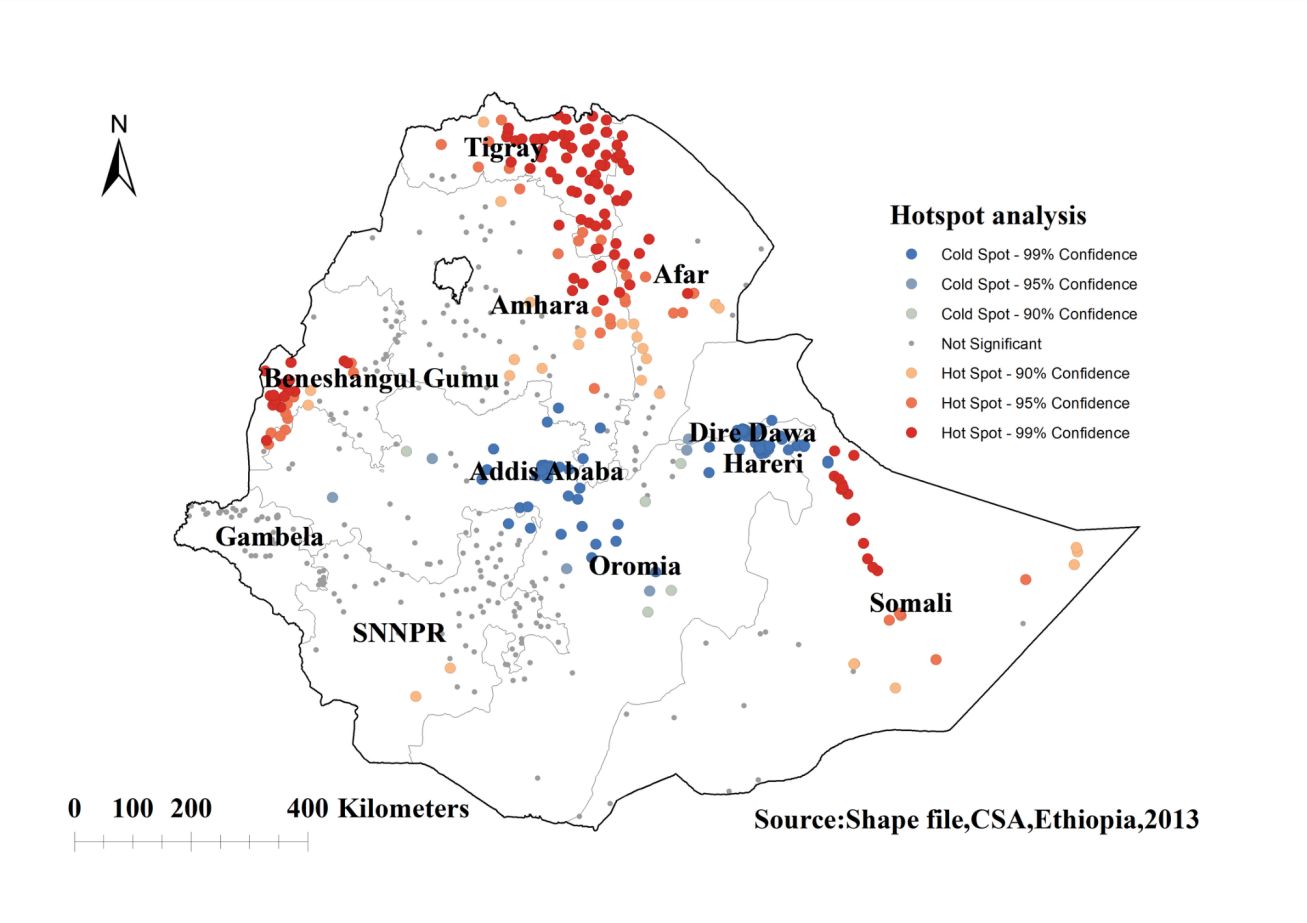
Hot spot analysis of knowledge of the ovulatory cycle among young women in Ethiopia: EDHS 2016

We found significant spatial variability in the distribution of incorrect knowledge of the ovulatory period among young Ethiopian women, with significant hotspots found in Somalia, the Tigray region, Benishangul Gumuz, Northern Amhara, and some parts of the Afar regions of Ethiopia. The significant cold spot regions of incorrect knowledge of the ovulatory period among young women were detected in the Addis Ababa, Dire Dawa, Harari, and Oromia regions of Ethiopia (Fig. 3).

#### Spatial interpolation of knowledge of the ovulatory period among young women in Ethiopia

**Fig. 4:**
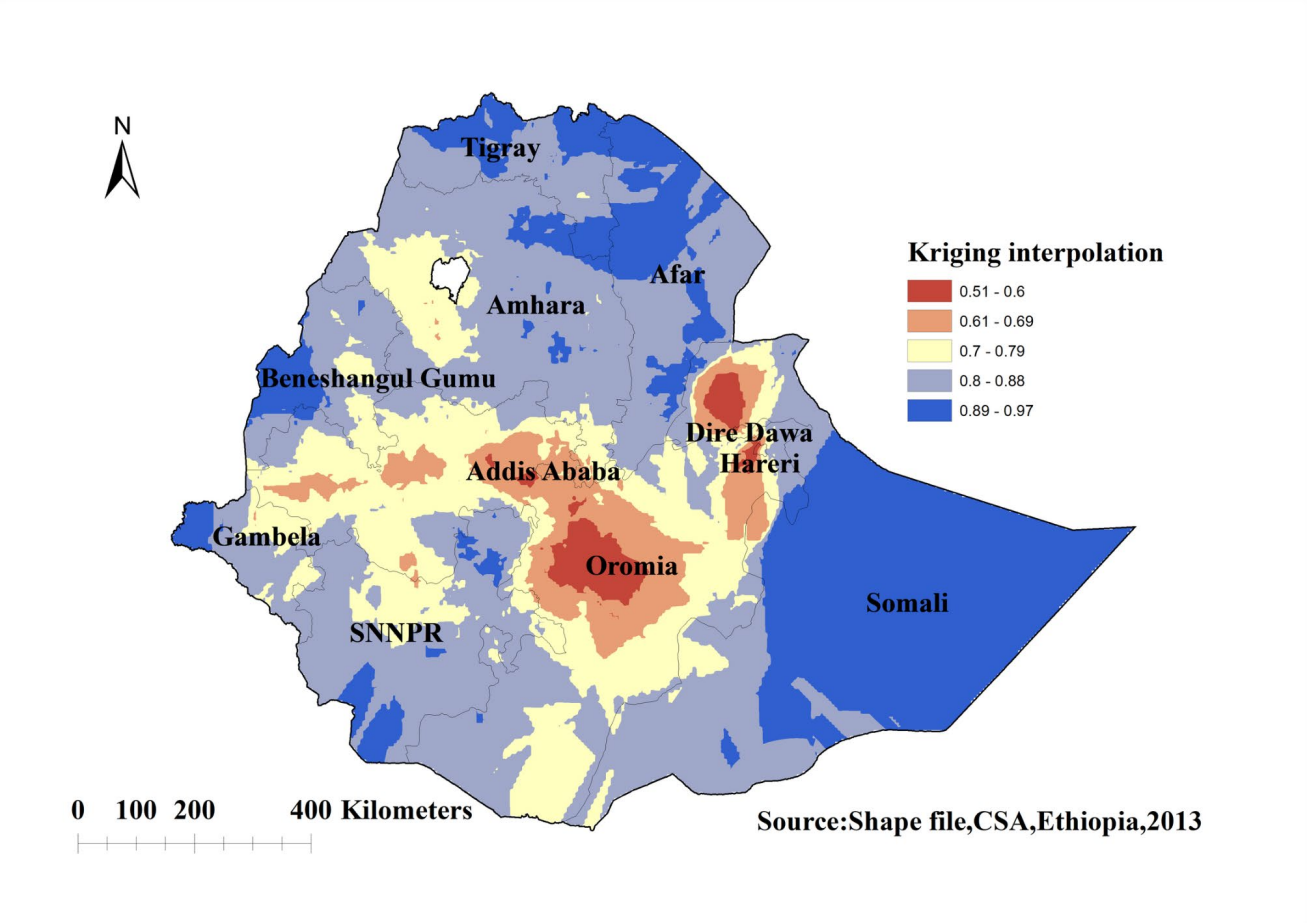
Kriging interpolation of knowledge of the ovulatory cycle among young women in Ethiopia: EDHS 2016

The highest predicted proportion of incorrect knowledge of the ovulatory period among young women was detected in Somalia, Afar, Tigray, the western regions of Benishangul gumuz and Gambella, the southern border of SNNPR, and the Northern Amhara regions of Ethiopia. The lowest predicted proportion of incorrect knowledge of the ovulatory period among young women was detected in the Addis Ababa, Dire Dawa, Harari, and Oromia regions of Ethiopia (Fig. 4).

#### Spatial scan statistical analysis of knowledge of the ovulatory period among young women in Ethiopia

**Fig. 5:**
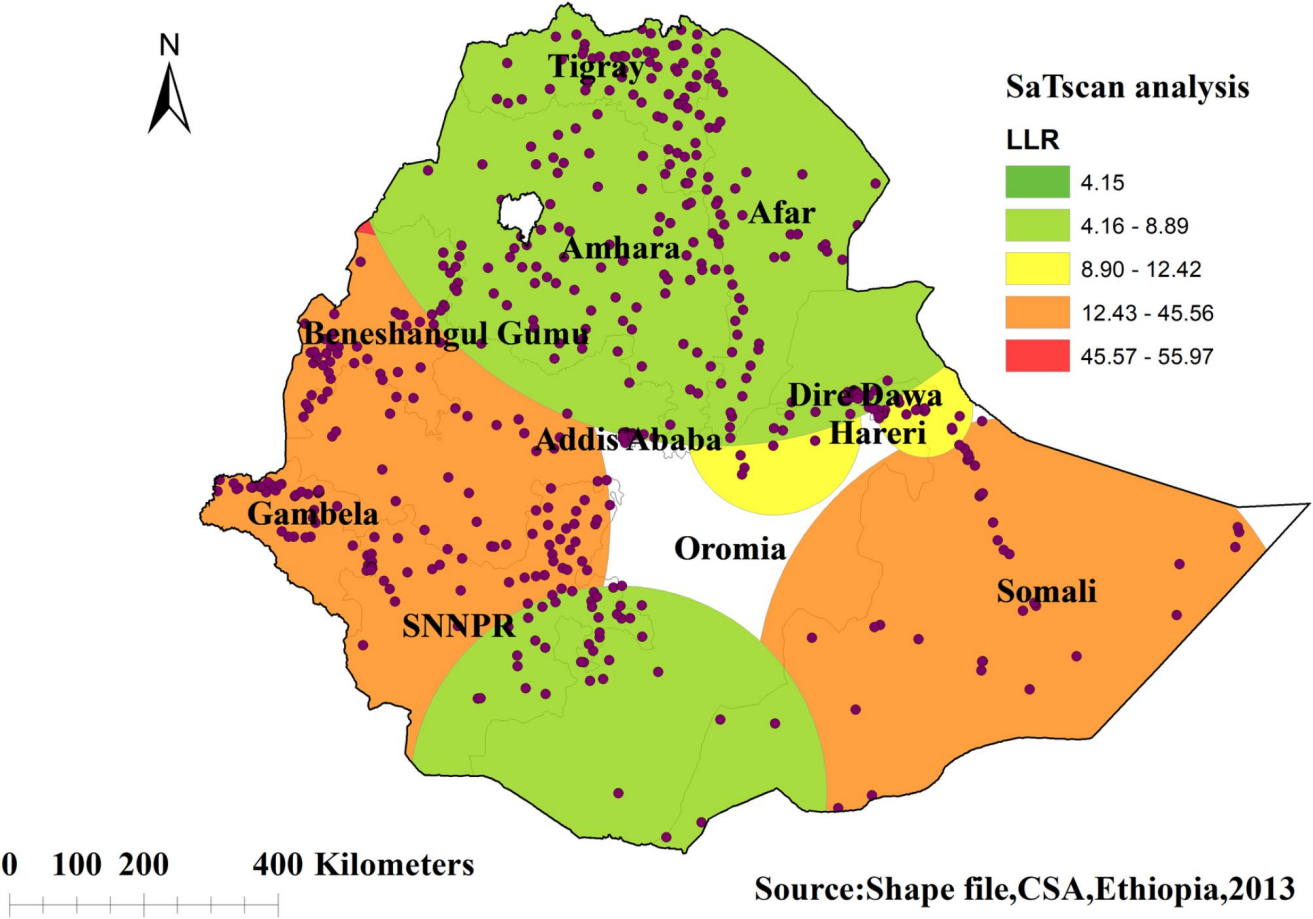
Spatial scan statistics analysis of knowledge of the ovulatory cycle among young women in Ethiopia: EDHS 2016

The SaTScan analysis result identified a significant 217 primary clusters of incorrect knowledge of the ovulatory period detected in regions of Somalia, SNNPR, Benishangul gumuz, and Gambella regions of Ethiopia at 14.390268 N, 37.773392 E with a 573.95 km radius, with a relative risk of 1.16 and log-likelihood ratio (LLR) of 55.96 at a p-value < 0.001 (Fig. 5).

### Fixed effect analysis results

Based on the final model result, the age of the youth, educational status of the women, educational status of the husband, and current use of contraceptives were found to be significantly associated with having knowledge of the ovulatory period among young women in Ethiopia.

Youths aged 20–24 years were found 1.98 [AOR = 1.98; 95% CI; 1.46, 2.70] times more likely to be knowledgeable about ovulation compared with the youth in the age group of 15–19 years. The odds of having good knowledge of the ovulatory period among youths who have primary education is 1.70 [AOR = 1.70; 95% CI; 1.23, 2.35] higher than youths who have no education. The odds of having good knowledge of the ovulatory period among youths who have secondary & higher were 2.30 times higher than youths who have no education [AOR = 2.30; 95% CI; 1.41, 3.74]. The odds of having good knowledge of the ovulatory period among youths whose husbands have primary education is 39% [AOR = 1.39; 95% CI; 1.02, 1.91] higher than youths whose husbands have no education. The odds of having good knowledge of the ovulatory period among youths who use contraception were increased by 66% [AOR = 1.66; 95% CI; 1.24, 2.22] than youths who do not use contraception (Table 3).

**Table 3:** Multilevel analysis of factors associated with knowledge of the ovulatory period among young women, 2016 EDHS

Variables	**categories**	Null model	Model I	Model II	Model III
			AOR [95% CI]	AOR [95% CI]	AOR [95% CI]
Age of women	15–19		1.00		1.00
	20–24		2.08 [1.53, 2.83]		1.98 [1.46, 2.70]***
Marital status	Not married		1.00		1.00
	Married		1.00 [0.50, 1.98]		1.13 [0.57, 2.26]
Women education status	No education		1.00		1.00
	Primary		1.70 [1.24, 2.34]		1.70 [1.23, 2.35]***
	Secondary and higher		2.23 [1.39, 3.57]		2.30 [1.41, 3.74]***
Husband education status	No education		1.00		1.00
	Primary education		1.40 [1.02, 1.91]		1.39 [1.02, 1.91]**
	Secondary & higher education		1.13 [0.74, 1.73]		1.05 [0.68, 1.61]
Current working status	Not working		1.00		1.00
	Working		0.79 [0.61, 1.03]		0.81 [0.62, 1.05]
Media exposure	No		1.00		1.00
	Yes		1.25 [0.93, 1.68]		1.13 [0.83, 1.53]
Contraceptive use	No		1.00		1.00
	Yes		1.67 [1.26, 2.22]		1.66 [1.24, 2.22]***
Menstruated in the last six weeks	No		1.00		1.00
	Yes		0.94 [0.72, 1.22]		0.93 [0.71, 1.20]
Wealth index	Poor		1.00		1.00
	Middle		0.99 [0.69, 1.42]		0.91 [0.63, 1.31]
	Rich		1.24 [0.87, 1.77]		0.90 [0.60, 1.34]
**Community level variables**					
Residence	Rural			1.06 [0.75, 1.50]	1.23 [0.64, 2.37]
	Urban			1.00	1.00
Community level poverty	Low			1.00	1.00
	High			0.85[0.63, 1.13]	0.73 [0.42, 1.25]
Community level of media exposure	Low			1.00	1.00
	High			1.84 [1.38, 2.45]	1.65 [0.98,2.78]
Community level of women’s education	Low			1.00	1.00
	High			1.23 [0.95, 1.58]	0.87 [0.55, 1.39]
Region	Somalia			1.00	1.00
	Tigray			0.99[0.51, 1.93]	0.50 [0.18, 1.38]
	Afar			1.18 [0.42, 3.28]	0.70 [0.16, 3.00]
	Amhara			1.89 [1.02, 3.47]	0.92 [0.36, 2.36]
	Oromia			3.70 [2.03, 6.75]	2.09 [0.86,5.08]
	Benishangul			1.01 [0.37, 2.71]	0.27 [0.04, 1.49]
	SNNPR			1.54 [0.82, 2.87]	0.81 [0.31, 2.06]
	Gambella			1.76 [0.47, 6.59]	0.76 [0.09, 5.99]
	Harari			3.80 [1.10, 13.12]	2.53 [0.35, 18.27]
	Addis Ababa			2.57 [1.31, 5.04]	1.41 [0.43,4.57]
	Dire Dawa			2.72 [1.03, 7.15]	1.50 [0.28, 8.05]

AOR = adjusted odds ratio; CI = confidence interval. * = P-value < 0.05, ** = Pvalue < 0.01, *** = Pvalue < 0.001

## Discussion

The purpose of this study was to assess the spatial variations and determinants of knowledge of the ovulatory period among young women in Ethiopia. The spatial pattern of incorrect knowledge of the ovulatory period varied across Ethiopian regions. Significant hotspot areas (high proportion) of incorrect knowledge of the ovulatory period were identified in Somalia, Tigray, Benishangul Gumuz, Northern Amhara, and some parts of the Afar regions of Ethiopia. Statistically significant primary clusters of incorrect knowledge of the ovulatory period were found in Ethiopia Somalia, SNNPR, Benishangul gumuz, and Gambella regions of Ethiopia. Most of these regions with a high proportion of incorrect knowledge of the ovulatory period are pastoralist regions. The possible reason might be that youths living in these pastoralist regions are most likely to be uneducated and they have less chance to be media exposed [41]. The findings emphasize the need of conducting location-specific interventions by the concerned bodies.

In the final model result, the age of the youth, educational status of the women, educational status of the husband, and current use of contraceptives were found to be significantly associated with having knowledge of the ovulatory period among young women in Ethiopia. Being older youth has a positive association with correct knowledge of the ovulatory period than youths aged 15–19. This finding is consistent with a study done in Ethiopia [4], and the USA [26]. The possible explanation is that older women are more likely to be exposed to reproductive health issues and may have been exposed to health information.

Youths having education and having educated husbands have a positive impact on their knowledge of the ovulatory period. The finding is supported by other studies done in Ethiopia [4, 20, 22], Sub-Saharan Africa [5, 22], and Uganda [42]. The possible reason is that educated women have access to information about the ovulatory period. As a woman’s or her partner’s education level rises, their awareness of reproductive system physiology will also be increased [20].

Youths who are currently using contraception were more likely to be aware of their ovulatory period than non-users. The result is in line with other studies done in Ethiopia [4], sub-Saharan Africa [5], and Ghana [43]. The possible reason might be that women who use contraception have access to family planning counseling, including natural family planning services.

This study used a nationally representative large, weighted dataset with an advanced statistical model that considers the data’s hierarchical structure. Furthermore, we conducted a spatial analysis and identified significant hotspot areas where incorrect knowledge of the ovulatory cycle was prevalent. However, the data used in this study are cross-sectional data, which limits the conclusions about the causality of factors in the dependent variable. As this survey is based on self-reported data from respondents, there is a potential for recall bias, given that individuals were asked to recall past events. Since we used a secondary data, information about some independent variables of a determinant of knowledge of the ovulatory cycle was missed.

## Conclusion

There was a non-random spatial pattern in the distribution of knowledge of the ovulation period among young women in Ethiopia. Age of youth, educational status, education of husband, and contraceptive use were significant predictors of knowledge of the ovulatory period among young women in Ethiopia. Hence, interventions should prioritize at-risk youths residing in regions with limited knowledge of the ovulatory period to enhance their awareness of the fertility window.

## Data Availability

The Ethiopian Demographic and Health Survey 2016 data can be accessed from the Measure DHS program at http://www.measuredhs.com/data.

## Abbreviations

AOR: Adjusted Odds Ratio
CI: Confidence Interval
COR: Crude odds ratio
DHS: Demographic and Health Survey
EAs: Enumeration Areas
EDHS: Ethiopian Demographic and Health Survey
ICC: Intra Class Correlation
KOC: Knowledge of ovulatory cycle
MOR: Median Odd Ratio
SNNP: Southern nation nationalities of people
VIF: Variance inflation factor
